# Huanglian Jiedu Decoction Treats Ischemic Stroke by Regulating Pyroptosis: Insights from Multi-Omics and Drug–Target Relationship Analysis

**DOI:** 10.3390/ph18060775

**Published:** 2025-05-23

**Authors:** Yixiao Gu, Zijin Sun, Tao Li, Xia Ding

**Affiliations:** 1 School of Traditional Chinese Medicine, Beijing University of Chinese Medicine, Beijing 100029, China; 20220931101@bucm.edu.cn (Y.G.); 20210931013@bucm.edu.cn (Z.S.); 2 Shaanxi Key Laboratory of TCM Encephalopathy, Shaanxi University of Chinese Medicine, Xianyang 712046, China

**Keywords:** HLJD, pyroptosis, ischemic stroke, myeloid cell, multiomics, drug target relationship

## Abstract

**Background:** Ischemic stroke (IS) is a severe condition with limited therapeutic options. Pyroptosis, a type of programmed cell death linked to inflammation, is closely associated with IS-related damage. Studies suggest inflammation aligns with the traditional Chinese medicine (TCM) concept of “fire-heat syndrome”. Huanglian Jiedu Decoction (HLJD), a TCM formula known for clearing heat and purging fire, has shown therapeutic effects on IS, potentially by regulating pyroptosis. Study design: Eight-week-old male mice were divided into six groups: sham operation, model, positive drug, and low-, medium-, and high-dose HLJD groups. After a week of adaptive feeding, mice received respective treatments for five days, followed by modeling on the sixth day, with samples collected 23 h post-perfusion. Analyses included multi-omics, physiology, histopathology, virtual drug screening, target affinity assessment, and molecular biology techniques to measure relevant indicators. **Results:** HLJD effectively mitigated IS-related damage, maintaining neurological function, reducing ischemic levels, protecting cellular morphology, inhibiting neuronal apoptosis, and preserving blood–brain barrier integrity. Bioinformatics of high-throughput omics data revealed significant activation of pyroptosis and related inflammatory pathways in IS. ScRNA-seq identified neutrophils, macrophages, and microglia as key pyroptotic cell types, suggesting potential therapeutic targets. Network pharmacology and molecular docking identified NLRP3 as a critical target, with 6819 ligand–receptor docking results. SPR molecular fishing, LC-MS, molecular dynamics, and affinity measurements identified small molecules with high affinity for NLRP3. Molecular biology techniques confirmed that HLJD regulates pyroptosis via the classical inflammasome signaling pathway and modulates the inflammatory microenvironment. **Conclusions:** Following IS, pyroptosis in myeloid cells triggers an inflammatory cascade, leading to neural damage. HLJD may inhibit NLRP3 activity, reducing pyroptosis and associated inflammation, and ultimately mitigating damage.

## 1. Introduction

Stroke is the second most common cause of death after ischemic heart disease and a leading cause of disability worldwide. It is considered one of the most prevalent and devastating diseases affecting humanity today [1]. According to the Global Burden of Disease Report, the number of patients diagnosed with stroke has been steadily increasing in recent years, leading to significant economic burdens, especially in low- and middle-income countries [2]. Statistics show that nearly 6.5 million people died from stroke in 2013, and it is estimated that by 2030, stroke will cause 12 million deaths [3]. Therefore, elucidating the mechanisms underlying ischemic brain injury is of paramount importance. In this study, we explored the biological processes involved in an attempt to identify potential therapeutic targets and to provide some preliminary research basis for the development of effective treatments.

Pyroptosis is a novel form of programmed cell death triggered by the activation of inflammatory Caspase-1 by various inflammasomes. This process mediates the action of the Gasdermin (GSDM) D protein, leading to cell lysis and the release of cytoplasmic contents and pro-inflammatory mediators, including IL-1β and IL-18, ultimately resulting in an excessive inflammatory response [4,5]. Specifically, NLR family pyrin domain containing 3 (NLRP3) is considered one of the primary inflammasomes, highly expressed in the brain due to its crucial role in recognizing cellular damage and initiating inflammatory cascades, ultimately leading to cell death [6]. Some studies indicate that the NLRP3 inflammasome plays a crucial role in the occurrence of brain ischemia/reperfusion (I/R) injury [7]. Pyroptosis serves as an effective inducer of the pro-inflammatory pathway in IS, primarily distributed in the ischemic penumbra [8]. Recent studies suggest that pyroptosis may be associated with brain I/R injury, involving processes such as free radical generation, inflammation, autophagy, and mitochondrial dysfunction. According to reports, intravenous immunoglobulin therapy targeting Caspase-1 can inhibit the activity of the NLRP3 inflammasome, thereby exerting neuroprotective effects in experimental stroke models [9]. Another study indicates that a deficiency of the NLRP3 gene can protect mice from ischemic injury [10,11].

HLJD is a traditional Chinese medicinal formula known for its heat-clearing and detoxifying properties. Its composition includes *Coptidis chinesis Franch* (Huang-Lian), *Scutellaria baicalensis Georgi* (Huang-Qin), *Phellodendron amurense Rupr* (Huang-Bo), and *Gardenia jasminoides Ellis* (Zhi-Zi), in a ratio of 3:2:2:3. Emerging studies suggest that HLJD and its constituents and extracts possess pharmacological effects against inflammation, infectious diseases, neurological disorders, and certain cardiovascular diseases [12,13,14]. Substantial evidence has established the correlation between HLJD and inflammatory injury [15,16]. However, due to the absence of appropriate methodologies and the multi-target, multi-pathway nature of traditional Chinese medicine, there is an abundance of information that complicates the extraction of pivotal insights. The pharmacological mechanisms underlying HLJD’s effects on IS remain incompletely understood.

This study employs a multi-omics approach to unravel the intricate interplay of pharmacodynamics, phenotype correlations, drug targets, and specific mechanisms. It seeks to elucidate whether HLJD can alleviate IS by modulating pyroptosis, aiming to uncover potential target molecules and pivotal pathways. This research endeavors to provide profound scientific insights into the mechanisms through which HLJD exerts therapeutic effects in IS.

## 2. Results

### 2.1. HLJD Mitigates Neural Damage and Enhances Perfusion in Mice with tMCAO

**Figure 1:**
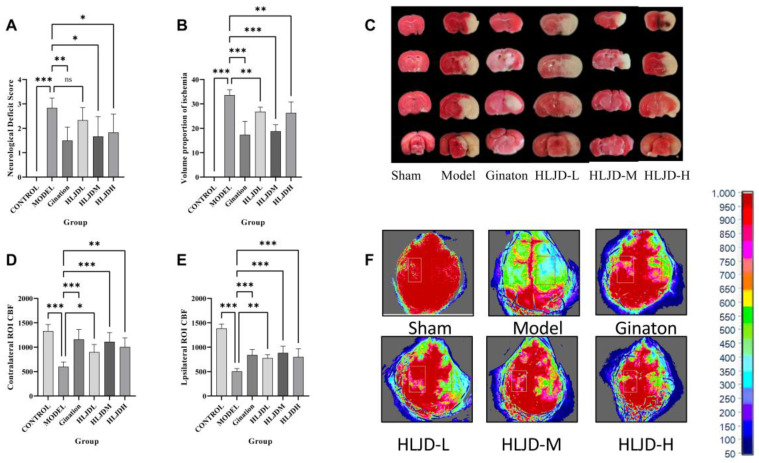
Protective effect of HLJD on cerebral infarction volume, cerebral blood flow, and nerve function defects in tMCAO mice. (**A**) Neurological deficit score analysis (Longa Score). (**B**) Detection of cerebral infarct volume. (**C**) The volume of cerebral infarction in mice by TTC Staining. (**D**) Healthy side CBF measurement. (**E**) CBF on the ischemic side was measured. (**F**) The CBF was measured by laser scattering. Results are presented as means ± SD. *n* = 6, one-way ANOVA, * *p* < 0.05, ** *p* < 0.01, *** *p* < 0.001.

As previously described, to investigate the protective effects of HLJD on I/R injury, we conducted various assessments 24 h after inserting the intraluminal suture. Neurological function deficits and TTC staining evaluations revealed significant increases in both the level of neurological deficits and the volume of the ischemic lesion in mice post-modeling. However, administration of Ginaton or HLJD significantly mitigated the neurological damage and reduced the ischemic lesion volume (Figure 1A–C). Laser speckle flow imaging showed that blood flow in both the contralateral and ipsilateral brains of mice decreased significantly after modeling, whereas HLJD administration partially restored blood flow levels (Figure 1D–F).

### 2.2. HLJD Reduces Cellular Damage, Neuronal Apoptosis, and Maintains Blood–Brain Barrier Integrity in Mice with tMCAO

**Figure 2:**
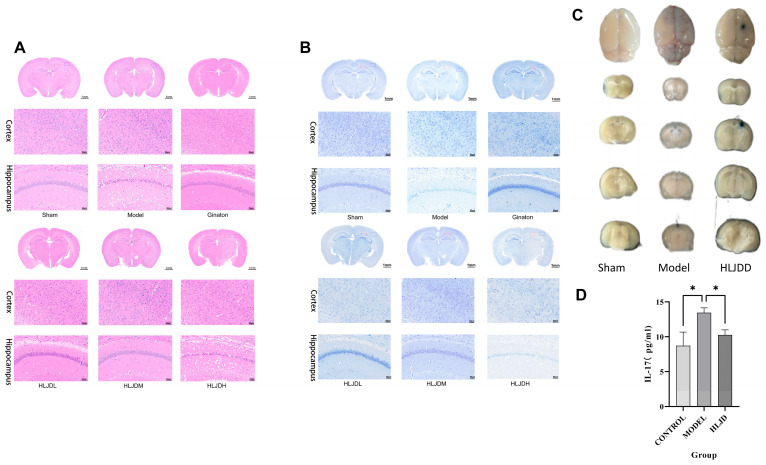
Effect of HLJD on micropathology and BBB permeability in tMCAO mice. (**A**) HE in the cortex and hippocampus of tMCAO mice. (**B**) Nissl staining in the cortex and hippocampus of tMCAO mice. (**C**) BBB was tested by EB. (**D**) EB absorbance measurement. Results are presented as means ± SD. *n* = 6, one-way ANOVA, * *p* < 0.05.

HE and Nissl staining observations demonstrated that HLJD treatment significantly improved the arrangement and morphology of brain cells in modeled mice, reduced ischemic pathological damage, and prevented the loss of Nissl bodies, indicating a good therapeutic effect on tMCAO mice (Figure 2A,B). The BBB permeability experiment further demonstrated reduced permeability in the HLJD group, protecting BBB function (Figure 2C,D). Next, we will further investigate the underlying mechanisms.

### 2.3. Activation of Pyroptosis and Associated Inflammatory Signaling Pathways in the Brain During Ischemic Stroke, with the Classical Inflammasome Pathway Playing a Key Role

**Figure 3:**
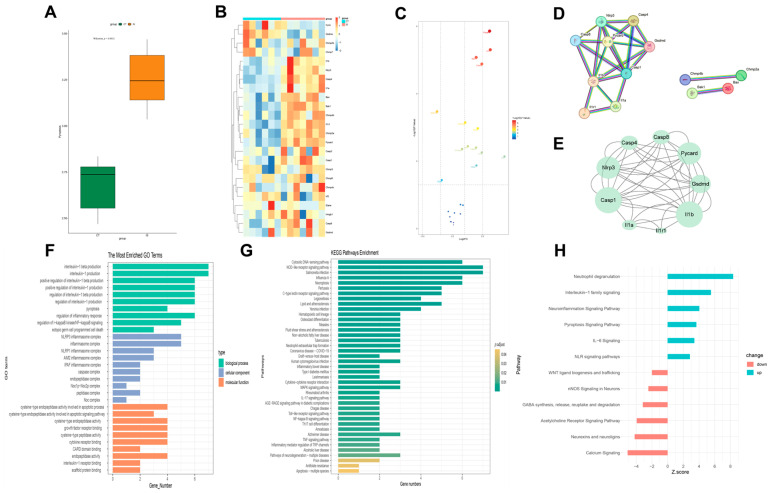
Analysis of bulk RNA-seq in tMCAO mice. (**A**) Observe the differences in pyroptosis gene set scores between the CT group and the IS group using the ssGSEA method. (**B**) Heatmap presentation of differential genes. (**C**) Volcano map presentation of differential genes. (**D**) PPI network formed by 14 differential genes. (**E**) The core pyroptosis gene network in IS mice. (**F**) GO analysis of differential genes. (**G**) KEGG analysis of differential genes. (**H**) IPA analysis of differential genes.

To explore the role of pyroptosis in IS, as well as identify key targets and cell types, we utilized transcriptomics approaches. From literature review and GSEA database mining, we compiled a gene set of 24 pyroptosis-related genes (Supplementary Table S1). The score of this gene set in the normal group and the MCAO group was observed. The scoring shows statistically significant differences and suggests activation of pyroptosis post-IS (Figure 3A). For differential analysis, genes with |LOGFC| ≥ 0.15 from the target gene set were selected, yielding 14 intersecting genes. These were identified as key genes between control and model groups, presented in a volcano plot and heatmap (Figure 3B,C).

The 14 differentially expressed genes are constructed into an interaction network, revealing three main interaction clusters (Figure 3D). This network is further analyzed using Cytoscape, resulting in a core interaction network containing 9 nodes and 44 edges (Figure 3E), using the following parameters: Degree cutoff = 2, K-Core = 2, Node Score Cutoff = 0.2, Max. Depth = 100, and Score threshold = 5.5.

GO enrichment analysis of the 9 core genes revealed 651 BP pathways, 21 CC pathways, and 48 MF pathways. We displayed 10 pathways each for BP, CC, and MF (Figure 3F), including pyroptosis, interleukin-1 production, and the NLRP3 inflammasome complex. KEGG analysis identified 41 pathways, including NOD-like receptor signaling, necroptosis, neurodegeneration pathways, and NF-kappa B signaling (Figure 3G).

The IPA results indicated the activation (Z-score > 2) of pathways such as neuroinflammation signaling, pyroptosis signaling, and NLR signaling in the model group. Conversely, pathways like GABA synthesis/release/reuptake/degradation, acetylcholine receptor signaling, neurexins and neuroligins, and calcium signaling were inhibited (Z-score < −2) (Figure 3H). Analysis of the pyroptosis signaling pathway suggested possible misannotation of IL-18 and GSDME as downregulated; thus, these genes were targeted for further validation (Supplementary Figure S1). Validation experiments were conducted to confirm the expression levels of IL-18 and GSDME.

This comprehensive analysis indicates that pyroptosis is activated following IS, primarily involving the classic NLRP3-associated pyroptosis pathway and related genes. Based on these findings, we will continue to investigate the cellular heterogeneity of pyroptosis phenotypes post-IS to further understand its role in IS pathology.

### 2.4. Disruption of the Blood–Brain Barrier and Infiltration of Peripheral Immune Cells into the Central Nervous System Following Ischemic Stroke

**Figure 4:**
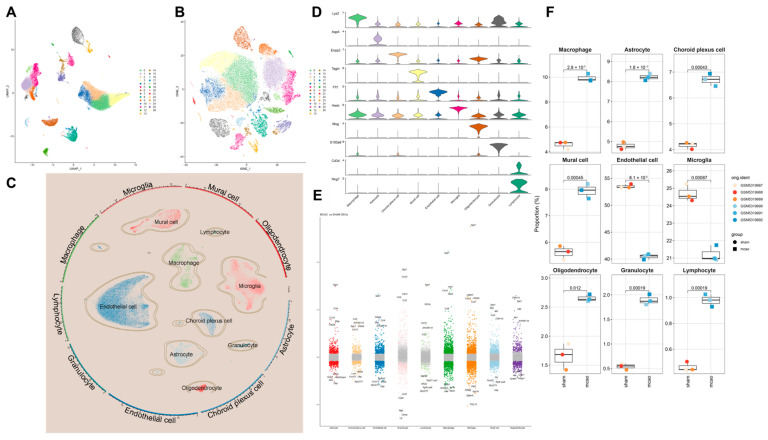
Analysis of scRNA-seq in tMCAO mice. (**A**) UMAP plot after dimensionality reduction and clustering. (**B**) T-SNE plot after dimensionality reduction and clustering. (**C**) UMAP plot showing cell type annotations after clustering. (**D**) MARKER genes of each cell type. (**E**) Differential genes of each cell type. (**F**) Proportional changes in each cell type.

To determine cellular heterogeneity in pyroptosis during IS and identify which cell types play major roles in the pyroptosis process, we performed scRNA-seq analysis. The clustering and dimensional reduction were visualized in two-dimensional space using both t-SNE and UMAP algorithms, showing the distribution of cell clusters (Figure 4A,B, Supplementary Figures S2 and S3).

Using marker genes from databases and the literature, we annotated nine cell types: Macrophages (Lyz2 high expression), Astrocytes (Aqp4 high expression), Choroid plexus cells (Enpp2 high expression), Mural cells (Tagln high expression), Endothelial cells (Flt1 high expression), Microglia (Hexb high expression), Oligodendrocytes (Mog high expression), Granulocytes (S100a8 high expression), and Lymphocytes (Cd3d and Nkg7 high expression) (Figure 4C,D). Differential gene expression analysis was conducted for each cell type, highlighting the top five upregulated and downregulated genes. Post-stroke, there was a significant increase in chemokine and cytokine levels across all cell types, indicating that tMCAO induced the release of chemokines to recruit peripheral immune cells, thus exacerbating inflammatory damage (Figure 4E,F).

These findings indicate that disruption of the blood–brain barrier and chemokine signaling facilitate infiltration of peripheral immune cells, thereby contributing to the post-IS inflammatory response within the brain. These findings highlight the cellular heterogeneity in pyroptosis during IS. Post-MCAO, there is an activation of inflammatory pathways and significant changes in cell population dynamics, with peripheral immune cells playing a crucial role in the ensuing brain inflammation.

### 2.5. Enhanced Upregulation of Pyroptotic Phenotype in Myeloid Cells: Microglia, Neutrophils, and Macrophages Following Ischemic Stroke

**Figure 5:**
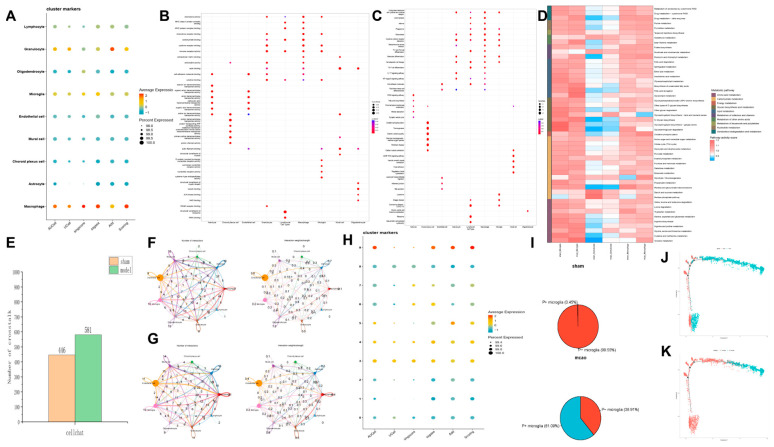
Subpopulation analysis of microglia, macrophages, and neutrophils. (**A**) Scores of the pyroptosis gene set in various cell types. (**B**) GO analysis of different genes in various cells. (**C**) KEGG analysis of different genes in various cells. (**D**) Metabolic analysis of microglia, macrophages and neutrophils. (**E**) The number of cell communications. (**F**) Cell communication in sham group. (**G**) Cell communication in m group. (**H**) Proportion of the number of microglia subsets in each group. (**I**) Pyroptosis gene set scores in subtypes of microglial cells. (**J**) The ratio of P- microglia and P+ microglia in sham group and mcao group. (**K**) Distribution of microglia in sham group and mcao group on the pseudo-time axis.

Using the pyroptosis gene set, we scored each cell type to determine pyroptosis activation levels. The top three cell types with the highest pyroptosis gene enrichment scores were macrophages, neutrophils, and microglia (Figure 5A, Supplementary Table S2). GO and KEGG enrichment analyses revealed that, compared to other cell types, macrophages, neutrophils, and microglia were enriched in pathways closely related to immune and inflammatory responses, such as immune receptor activity, cytokine receptor binding, chemokine receptor binding, the interleukin-17 signaling pathway, and the NF-κB signaling pathway (Figure 5B,C).

GO and KEGG enrichment analysis showed that inflammation-related pathways in microglia, neutrophils, and macrophages were significantly activated (Supplementary Figure S4).

Metabolic analysis of these three cell types revealed distinct metabolic pathway changes: Microglia: Significant changes in lipid metabolism, glycan biosynthesis, energy metabolism, and carbohydrate metabolism, particularly glycolysis and pentose phosphate pathway. Neutrophils: Metabolic changes were widespread, with almost all pathways shifting from a relatively suppressed state to an active state. Macrophages: Similar to microglia, with notable activation in glycolysis and pentose phosphate pathways (Figure 5D). Previous studies have shown that activated pro-inflammatory phenotypes of macrophages, microglia, and neutrophils rely primarily on glycolysis for rapid energy acquisition, supporting the observation that these cells are activated toward a pro-inflammatory state.

Using the CellChatDB database, we calculated cell communication networks. The normal group had 446 communication pairs, while the model group increased to 581 pairs (Figure 5E, Supplementary Figure S5).

Increased cell communication was observed among macrophages, neutrophils, and microglia post-IS, with a significant rise in interactions among these cells, indicating their active role in mediating disease progression (Figure 5F,G).

Re-clustering of microglia into 10 subgroups revealed that clusters 2, 3, 4, 5, 7, and 9 were more prevalent in the model group, whereas clusters 0, 1, 6, and 8 were predominant in the normal group (Figure 5H). Pyroptosis scoring showed that clusters 3, 4, 5, and 9 were positively expressed for pyroptosis (P+ microglia), while clusters 0, 1, 2, 6, 7, and 8 were negatively expressed (P- microglia) (Figure 5I). Proportion analysis demonstrated a significant increase in P+ microglia in the model group compared to the sham group, indicating an elevation in pyroptosis-positive microglia post-stroke (Figure 5J). Pseudotime analysis revealed a transition from P- microglia to P+ microglia as the condition shifted from the sham group to the model group (Figure 5K,L). Applying the same analysis to macrophages and neutrophils yielded similar results, with both cell types showing a shift toward pyroptosis activation post-IS (Supplementary Figures S6 and S7).

These analyses demonstrate that macrophages, neutrophils, and microglia play significant roles in pyroptosis during IS, with increased activation and metabolic changes reflecting their pro-inflammatory state. Enhanced cell–cell communication among these cells further underscores their involvement in IS pathology.

### 2.6. Neuroprotective Effects of HLJD via Inhibition of NLRP3-Mediated Pyroptosis

Through comprehensive data collection, a total of 1150 unique small molecules related to HLJD were compiled. Based on our prior findings, we focused on classical inflammasome signaling pathway genes associated with pyroptosis. Combining these results with key gene data, we selected NLRP3, ASC, Caspase-1, Caspase-3, Caspase-4, Caspase-8, IL-1β, IL-18, and GSDMD as potential targets for further analysis. During the data download process, a total of 781 small molecules were obtained, with the specific details of the selected proteins available in Supplementary Table S3.

After docking small-molecule ligands with protein receptors, we generated 7029 combinations, successfully achieving 6819 protein–ligand docking results. In practical applications, binding energy is often used to evaluate the affinity between receptors and ligands. It is generally considered that binding energies less than −7.0 kcal/mol (1 kcal = 4.2 kJ) indicate strong binding activity, respectively. The lower the binding energy, the higher the affinity and stability of the receptor–ligand conformation. Our study showed that there were 6130 receptor–ligand pairs and 2590 pairs with ≤−7.0 kcal/mol, indicating that most receptor–ligand pairs have good binding potential.

**Figure 6:**
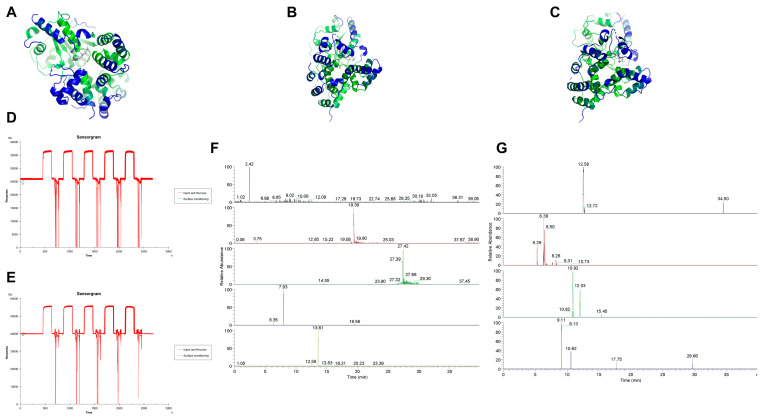
Molecular docking mode, SPR fishing diagram, and LC-MS diagram. (**A**) NLRP3-Kihadanin A docking diagram. (**B**) NLRP3-Kihadanin B docking diagram. (**C**) NLRP3-Obacunone docking diagram. (**D**) SPR fishing combined resonance unit performance chart with blank chip control. (**E**) NLRP3 protein binding chip control SPR fishing binding resonance unit performance map. (**F**) LC-MS cationic material diagram. (**G**) LC-MS anionic material diagram.

The top three receptor–ligand complexes in terms of binding energy were NLRP3-Kihadanin A, NLRP3-Kihadanin B, and NLRP3-Obacunone (Figure 6A–C). The top 10 receptor–ligand binding energy results are shown in Table 1. Among these combinations, the receptors were primarily NLRP3 and IL-18, with 9 out of 10 being NLRP3. This suggests that NLRP3 is likely the main target for binding in the regulation of IS-induced pyroptosis by HLJD.

Using SPR fishing analysis with chips containing NLRP3 and blank controls, we measured the affinity levels between HLJD and NLRP3. The affinity of HLJD for NLRP3 was significantly higher compared to the blank control, confirming that HLJD contains small molecules that can bind to NLRP3 with a certain degree of affinity (Figure 6D,E). After background subtraction, we performed spectral analysis and identified four small molecules with potential affinity for NLRP3 in the anion mode: Chrysin-7-O-Glucuronide, Isomartynoside, Neochlorogenic Acid, and Puerarin. In the cation mode, we identified five molecules: Dictamine, Matrine, Rutin, Skullcapflavone II, and Wogonoside (Figure 6F,G).

**Figure 7:**
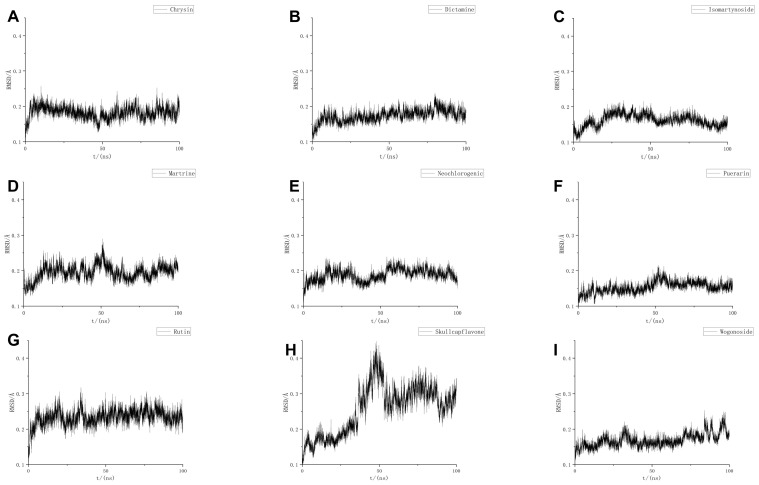
NLRP3 molecular dynamic binding of nine small molecules to RMSD determined by LC-MS. (**A**) RMSD of NLRP3-Chrysin-7-O-Glucuronide. (**B**) RMSD of NLRP3-Dictamine. (**C**) RMSD of NLRP3-Isomartynoside. (**D**) RMSD of NLRP3-Matrine. (**E**) RMSD of NLRP3-Neochlorogenic Acid. (**F**) RMSD of NLRP3-Puerarin. (**G**) RMSD of NLRP3-Rutin. (**H**) RMSD of NLRP3-Skullcapflavone II. (**I**) RMSD of NLRP3-Wogonoside.

MD calculations, including RMSD, RMSF, H-bond, SASA, Rg, and binding free energy, showed that all molecules except NLRP3-Skullcapflavone II could bind to NLRP3 and remain relatively stable (Figure 7A–I, Table 2, Supplementary Figures S8–S11).

**Figure 8:**
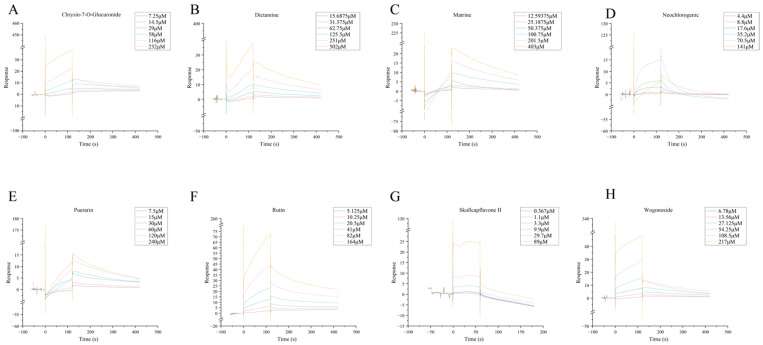
The affinity of NLRP3 to eight small molecules (available) derived from LC-MS detection was determined using SPR. (**A**) NLRP3-Chrysin-7-O-Glucuronide. (**B**) NLRP3-Dictamine. (**C**) NLRP3-Matrine. (**D**) NLRP3-Neochlorogenic Acid. (**E**) NLRP3-Puerarin. (**F**) NLRP3-Rutin. (**G**) NLRP3-Skullcapflavone II. (**H**) NLRP3-Wogonoside.

Next, we measured the affinity of the eight obtainable small molecules with NLRP3 using SPR. The results indicated that all eight small molecules exhibited affinity for NLRP3 and could inhibit its activity, thereby reducing pyroptosis activation and inflammatory damage (Figure 8A–H). However, based on the aforementioned molecular dynamics results, Skullcapflavone II showed relatively lower affinity for NLRP3, suggesting that further experimental validation may be necessary.

### 2.7. HLJD Reduces the Expression Levels of Molecules Associated with the Classical Pyroptosis Pathway

To validate our hypothesis, we conducted low-throughput molecular biology experiments, including RT-qPCR, Western Blot, and ELISA. In the classical inflammasome-related pyroptosis pathway, NLRP3 interacts with ASC and pro-Caspase-1 to form the NLRP3 inflammasome, which cleaves pro-Caspase-1 into Cleaved Caspase-1. Cleaved Caspase-1 can then cleave GSDMD, pro-IL-1β, and pro-IL-18 into their active forms, GSDMD-N, IL-1β, and IL-18, respectively. Therefore, we selected these targets for further validation.

**Figure 9:**
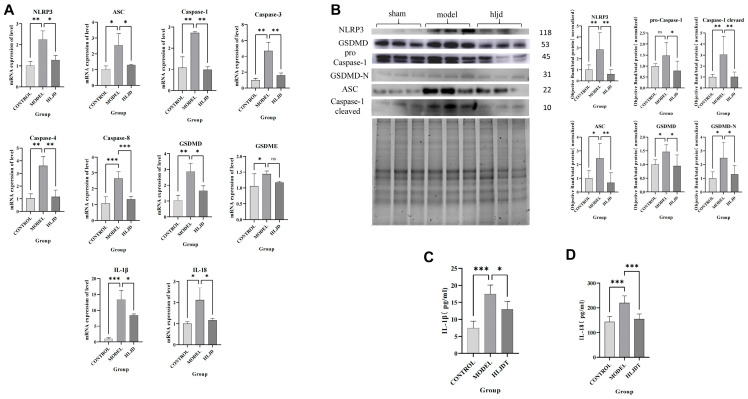
Pyroptosis-related mRNA and protein expression levels. (**A**) Pyroptosis-related mRNA expression levels. (**B**) WB plot and protein expression levels. (**C**) IL-1β protein expression levels. (**D**) IL-18 protein expression levels. Results are presented as means ± SD. *n* = 6, one-way ANOVA, * *p* < 0.05, ** *p* < 0.01, *** *p* < 0.001.

First, our RT-qPCR results (Figure 9A) showed a significant increase in the mRNA levels of the mentioned targets after IS model induction. Additionally, the mRNA levels of other pyroptosis-related targets, such as GSDME, Caspase-3, Caspase-4, and Caspase-8, were also elevated to varying degrees. In contrast, the mRNA levels of these targets were reduced in mice treated with HLJD compared to the MCAO model group.

Next, we performed WB and ELISA to assess the protein levels of the corresponding targets, obtaining similar results. In the model group, protein expression levels of NLRP3, ASC, pro-Caspase-1, Cleaved Caspase-1, full-length GSDMD, GSDMD-N, IL-1β, and IL-18 were significantly elevated compared to the normal group (Figure 9B–D), with pro-Caspase-1 showing an increase that was not statistically significant. Treatment with HLJD significantly reduced the expression levels of these proteins, indicating that HLJD administration could mitigate pyroptosis activation.

Interestingly, by comparing mRNA and protein expression levels, we observed that after HLJD treatment, both GSDMD and Caspase-1 RNA levels showed a downward trend, as did full-length GSDMD, GSDMD-N, pro-Caspase-1, and Cleaved Caspase-1 protein levels. Therefore, we propose that HLJD may reduce protein expression not only by inhibiting NLRP3 and the self-amplifying classical pyroptosis pathway but also by suppressing the translation of related targets. This effect is particularly evident in the reduction in full-length GSDMD and pro-Caspase-1 levels.

### 2.8. HLJD Modulates the Inflammatory Microenvironment, Reduces Pyroptosis Levels, and Exerts Neuroprotective Effects

**Figure 10:**
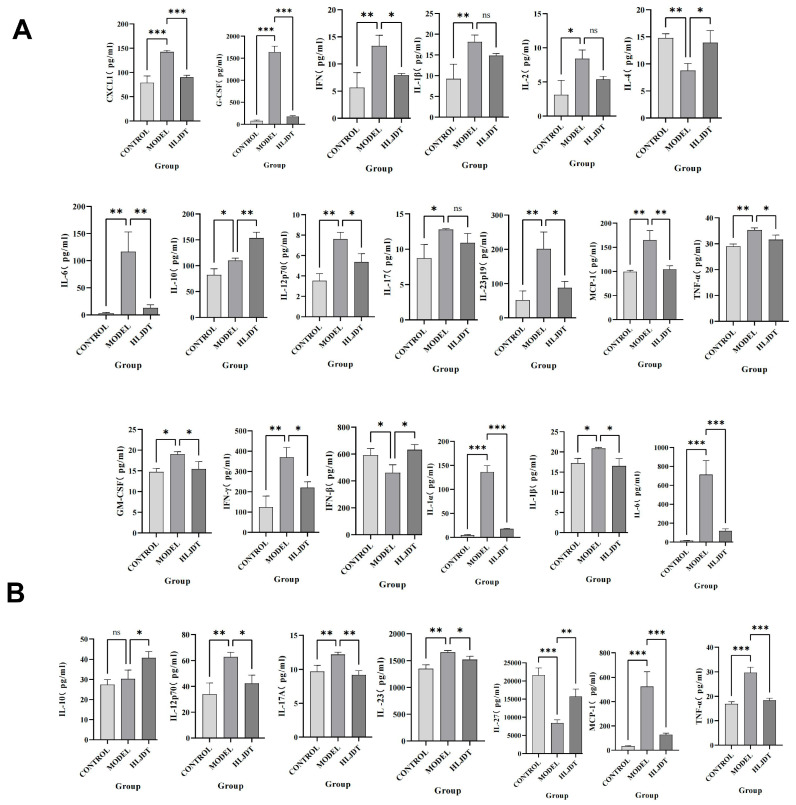
Expression levels of various inflammatory factors in blood and brain. (**A**) The expression level of a variety of inflammatory factors in blood. (**B**) The expression level of a variety of inflammatory factors in the brain. Results are presented as means ± SD. *n* = 3, one-way ANOVA, * *p* < 0.05, ** *p* < 0.01, *** *p* < 0.001.

As we expected, compared with the sham operation group, the levels of inflammatory factors in the model group were significantly increased (some of them were lower), and HLJD treatment could reverse the inflammatory damage caused by inflammatory factors to a certain extent (Figure 10A,B).

## 3. Discussion

IS poses a significant health risk with increasing incidence rates and a lack of effective clinical treatments, leading to substantial economic burdens. TCM attributes the pathogenesis of IS to the concept of “fire-heat” causing internal damage. Inflammation and inflammation-induced pyroptosis are critical factors in IS pathology, aligning closely with the TCM understanding of fire–heat mechanisms. In this study, we investigated the therapeutic effects and potential mechanisms of HLJD, a classic TCM formula known for its heat-clearing and detoxifying properties, in treating IS. Utilizing a multi-omics approach, we comprehensively explored how HLJD modulates pyroptosis to exert its protective effects against IS.

For a long time, TCM has been employed in treating neurological disorders [17]. Research has demonstrated that Buyang Huanwu Decoction can improve IS by regulating APRT and PED1B [18], while Tongqiao Huoxue Decoction can reduce IS damage by inhibiting ferroptosis through promoting ACSL4 ubiquitination [19].

The inflammatory response plays a key role in IS injury [20]. After the onset of IS, microglial cells in the brain are the first to become activated, leading to neuroinflammation and damage to the BBB. This activation results in the release of chemokines and cytokines. Peripheral innate immune cells, such as macrophages and neutrophils, are then recruited by these chemokines and enter the brain through the compromised BBB, causing further inflammatory damage.

Currently, substantial evidence indicates that inflammasome activation and pyroptosis play critical roles in the pathogenesis of IS [21,22]. In recent years, there has been an increasing number of studies focusing on the effects of TCM on these processes [23,24].

In previous studies, most attention has been focused on cell types within the brain, such as microglia, neurons, and endothelial cells [25,26,27,28]. Research on TCM has largely concentrated on the overall effects of complex formulas, often overlooking the specific small molecular compounds and the precise targets with which they interact [29,30,31,32].

However, in the disease process, the infiltration of peripheral immune cells (such as neutrophils and macrophages) into the central nervous system is also a critical factor in neuroinflammation and neurodamage. Additionally, inflammasomes are highly expressed in immune cells, making them key cell types in the process of pyroptosis. During the acute phase of IS, the primary activators and executors of immune responses are innate immune cells, including macrophages, neutrophils, microglia, and natural killer cells. CXCL1 and G-CSF facilitate neutrophil infiltration and promote their conversion to a pro-inflammatory phenotype. GM-CSF and IFN-γ attract and activate macrophages, leading to inflammatory damage. IL-1α, IL-1β, IL-6, IL-12p70, IL-17, IL-17A, IL-23, IL-23p19, MCP-1, and TNF-α are involved in various innate immune pathways and are closely related to inflammatory damage caused by monocytes, macrophages, neutrophils, and NK cells. Inhibiting their expression can reduce their activation and subsequent inflammatory damage to endothelial cells, neurons, and other cell types. Although IL-2 has a weaker association with innate immunity, it can enhance the cytotoxic effects of killer T cells, contributing to inflammatory damage. However, this effect is less pronounced in the acute phase of IS due to the delayed response of adaptive immunity compared to innate immunity. IFN-β, IL-4, and IL-10 can modulate monocytes toward an M2 macrophage phenotype, promoting protective and reparative effects. IL-27 does not directly repair or inhibit inflammation but can induce IL-10 production by stimulating T cells to express IL-12R, indirectly reducing neural and endothelial damage. These findings indicate that post-IS, the levels of pro-inflammatory cytokines in the brain are significantly elevated, and cytokines and chemokines that activate inflammatory cells, such as neutrophils and macrophages, also show increased levels. This suggests that the central–peripheral inflammatory microenvironment is activated toward a pro-inflammatory state following stroke. HLJD treatment not only suppresses pro-inflammatory cytokines and related chemokines but also increases anti-inflammatory cytokines like IL-10, promoting the polarization of macrophages, microglia, and neutrophils toward an anti-inflammatory phenotype (M2 and N2, respectively). This reduces inflammatory damage, promotes angiogenesis, and aids in the phagocytosis of debris, preventing further cellular damage. Consequently, HLJD modifies the inflammatory microenvironment and exerts a protective effect. Additionally, based on our single-cell results, pyroptosis is mainly activated in macrophages, neutrophils, and microglia. HLJD may reduce pyroptosis by decreasing the infiltration and inflammatory activation of these cells, thereby mitigating brain damage.

Therefore, using scRNA-seq analysis, we assessed the status of pyroptosis at the cellular level and identified that macrophages, neutrophils, and microglia are the main executors of pyroptosis during the acute phase of IS. Inhibiting pyroptosis-related pathways in these cells could potentially reduce neuroinflammation and provide neuroprotective effects. Interestingly, by comparing mRNA and protein expression levels, we observed that after HLJD treatment, both GSDMD and Caspase-1 RNA levels showed a downward trend, as did full-length GSDMD, GSDMD-N, pro-Caspase-1, and Cleaved Caspase-1 protein levels. Therefore, we propose that HLJD may reduce protein expression not only by inhibiting NLRP3 and the self-amplifying classical pyroptosis pathway but also by suppressing the translation of related targets. This effect is particularly evident in the reduction of full-length GSDMD and pro-Caspase-1 levels.

In this study, we employed a multi-omics approach, systematically progressing from pharmacodynamics and phenotypes to specific cells and small molecules/targets, to elucidate the mechanism by which HLJDT treats IS through the regulation of pyroptosis. By interpreting these findings from both macro and micro perspectives, we provide new insights into the pathological mechanisms of IS and identify potential therapeutic compounds. Furthermore, this methodology can serve as a model for investigating the relationship between traditional Chinese medicine formulas and diseases in future research.

However, our study has some limitations. In the animal experiments, we did not use NLRP3 inflammasome agonists or transgenic animals to conduct phenotype rescue experiments. In addition, due to the lack of commercial GSDMD-N or immunofluorescent antibody against GSDMD-N, we could not further validate the cell types that produce these pyroptosis phenotypes. These aspects require further analysis and exploration.

## 4. Materials and Methods

### 4.1. Materials and Reagents Used in the Experiment

*Coptidis chinesis Franch* (Huang-Lian) (220114004), *Scutellaria baicalensis Georgi* (Huang-Qin) (221128007), *Phellodendron amurense Rupr* (Huang-Bo) (220224001), and *Gardenia jasminoides Ellis* (Zhi-Zi) (220907001) were purchased from Beijing Qiancao Chinese Medicine Decoction Pieces Co., Ltd., (Beijing, China).

Chrysin-7-O-Glucuronide (TQ0287), Neochlorogenic Acid (T6S1538), Puerarin (T2815), Matrine (T2870), Dictamine (T5746), Rutin (T0795), and Wogonoside (T3318) were purchased from Target Mol (Shanghai, China). The NLRP3 protein (ABN-H00114548-P01-25 ug) was purchased from Abnova. The Series S Sensor Chip CM5 (29104988) was purchased from Cytiva (UCDP, Uppsala, Sweden). The primers were purchased from Beijing Dingguo Changsheng Biotechnology Co., Ltd., (Beijing, China). The compounds used in the manuscript are all above 99% pure.

TGX Stain-Free FastCast Kit (1610183) was purchased from BIO-RAD (Hercules, CA, USA). The antibodies against pro Caspase-1 + p10 + p12 (ab179515), GSDMD (ab219800), and NLRP3 (ab263899) were purchased from Abcam (Cambridge, UK). The Apoptosis-associated speck-like protein containing a CARD (ASC) antibody (67824S) was purchased from CST (Cell Signaling Technology) (Boston, MA, USA). The IL-1β (SEA563Mu) and IL-18 (SEA064Mu) ELISA kits were purchased from Cloud-Clone Corp (Wuhan, China). Proteins by Flow Cytometry Mouse LEGEND plex MU Proinflam Chemokine Panel (13-plex) (740150) was purchased from BioLegend (San Diego, CA, USA). Quantitative Measurement of 13 Mouse (FAM-INF-1-96 (96 tests)) was purchased from RayBiotech (Atlanta, GA, USA).

### 4.2. Preparation of HLJD

Weigh 150 g of *Coptidis chinensis* (Huang-Lian), 100 g of *Scutellaria baicalensis* (Huang-Qin), 100 g of *Phellodendron amurense* (Huang-Bo), and 150 g of *Gardenia jasminoides* (Zhi-Zi). Mix the four medicinal herbs thoroughly and divide them evenly into filtration bags. Add 5000 mL of water and let it soak for 1 h. Then, heat the mixture until it is boiling. Once boiling, reduce the heat to simmer and maintain a boil. Filter the mixture while hot using a 100-mesh filtration bag.

Add another 4000 mL of water and repeat the simmering process as before. Combine the liquids from both boilings and concentrate them. Divide the concentrated mixture into respective containers and dry it using a vacuum freeze dryer to obtain freeze-dried powder. Weigh the freeze-dried powder and calculate the extraction yield. The extraction rate of HLJD is 148.2 g/500 g = 29.64%

Daily dosage of HLJD: According to the calculation of a 60 kg adult, the dosage of raw drug is 30 g, that is, 30 g × 29.64%/60 kg × 12 = 1.7784 g/kg = 0.017784 g/10 g = 0.018 g/10 g daily. Calculate the volume of administration below. The gavage volume in mice is typically 0.2 mL/10 g. Dosing concentration = dosing volume/gavage volume, i.e., (0.018 g/10 g)/(0.2 mL/10 g) = 0.09 g/mL = 9 g/100 mL per mouse. Low dose: weigh 4.5 g HLJD and add animal drinking water to 100 mL. Medium dose: weigh 9 g of HLJD and add drinking water to 100 mL. High dosage: weigh 18 g HLJD and animal drinking water to 100 mL.

### 4.3. Design of the Animal Experiments

Eight-week-old male C57BL/6 J mice of specific pathogen-free grade, weighing 23 ± 2 g, were provided by Beijing Sibeifu Experimental Animal Technology Co., Ltd. The experimental animals were housed in a clean room at Beijing University of Chinese Medicine, with a 12-h light/dark cycle maintained at 25 ± 1 °C and 55 ± 10% humidity. The environment was free from noise interference.

This study was approved and supervised by the Animal Ethics Committee of Beijing University of Chinese Medicine, ensuring animal welfare throughout the experimental design and procedures. Ethics approval number BUCM-2024011901-1036.

In total, 48 male C57BL/6 J mice, aged 8 weeks and weighing 23 ± 2 g, were randomly divided into 6 groups using a random number table method, with 8 mice per group. The groups were as follows: sham surgery group, model group, Ginaton group, low-dose HLJD group, medium-dose HLJD group, and high-dose HLJD group.

After one week of acclimatization, the mice were administered intragastrically. The sham surgery and model groups received physiological saline. The Ginaton group received Ginaton solution prepared at a concentration of 2.16 mg/mL. The HLJD groups were dosed based on an adult equivalent of 60 kg: the low-dose group received a solution prepared from 15 g of freeze-dried powder per day, the medium-dose group received a solution from 30 g of freeze-dried powder per day, and the high-dose group received a solution from 60 g of freeze-dried powder per day.

In this study, we aimed to investigate the therapeutic mechanism of HLJD on acute ischemic stroke in mice. All groups received a dosage of 0.2 mL/10 g body weight/day for five consecutive days. On the sixth day, after the final dose administration, the transient middle cerebral artery occlusion (tMCAO) model was induced.

#### 4.3.1. Neurobehavioral Score

Starting from the insertion of the embolus into the middle cerebral artery, a 24-h countdown commenced. After this period, mice were removed from their housing cages and placed on an unobstructed flat experimental platform for Longa 5-point neurological behavioral assessment. This scale evaluates the severity of neurological damage, with higher scores indicating greater impairment.

#### 4.3.2. TTC

Starting from the insertion of the embolus into the middle cerebral artery, a 24-h countdown was initiated. After this period, the mice were euthanized, and their brain tissues were harvested for TTC staining.

#### 4.3.3. Detection of Cerebral Blood Flow (CBF)

Mice were anesthetized with isoflurane and fixed on a stereotaxic device. The skin on the top of the head was routinely disinfected, and afterward the scalp was cut. The meninges were then separated, and the skull between the coronal suture and the hermitage was fully exposed. After that, the cortical CBF was evaluated by laser speckle flow imaging.

#### 4.3.4. HE and Nissl Staining

The mouse brain tissues were fixed overnight in 4% paraformaldehyde and subsequently embedded in paraffin wax. Following embedding, the brain sections were stained using HE and Nissl staining solution.

#### 4.3.5. EB

To assess blood–brain barrier (BBB) permeability following tMCAO, transient middle cerebral artery occlusion modeling, EB staining was utilized. A 2% EB solution was prepared and injected into the tail vein of the mice 1 h prior to tissue collection. After 1 h of circulation, the mice were anesthetized and perfused transcardially with saline. The brains and brain tissue sections were photographed. The brain tissue was then homogenized, incubated at 54 °C for 2 h, and centrifuged. The optical density of the supernatant for each sample was measured at a wavelength of 620 nm.

### 4.4. Bulk RNA-Seq Data Preparation

Using the keywords “MCAO”, “ischemic stroke”, and “stroke”, datasets GSE116878 [33] and GSE227186 [34] were retrieved from the GEO database, selecting “mouse” as the species. Data processing was conducted using R version 4.1.3. The limma package was employed for within-dataset normalization to reduce batch effects related to sequencing depth and gene attributes, and gene expression levels were log2-transformed. Genes with |LOGFC| ≥ 0.15 were selected as target genes and considered differentially expressed genes (DEGs).

#### 4.4.1. Single Sample Gene Set Enrichment Analysis (ssGSEA)

Using the GSEA database and supported by a literature review, a pyroptosis gene set was obtained. The expression levels of these genes in each sample were scored using the ssGSEA method from the GSVA package. The differences between the control group and the ischemic stroke group were analyzed to observe if the target gene set showed overall differential expression between the two groups.

#### 4.4.2. Protein–Protein Interaction (PPI) Network Analysis and Enrichment Analysis

The obtained differentially expressed genes were imported into the STRING database with the species set to mouse and the minimum required interaction score set to 0.900 (highest confidence). The interaction results file was then imported into Cytoscape 3.9.1 for visualization analysis. The MCODE and CytoNCA plugins were used to perform topological analysis of the PPI network to identify the core interaction network. Using R and relevant R packages, Gene Ontology (GO) and Kyoto Encyclopedia of Genes and Genomes (KEGG) enrichment analyses and plotting were performed.

#### 4.4.3. Ingenuity Pathway Analysis (IPA)

IPA is a powerful tool for analyzing the direct interaction information between proteins, genes, compounds, cells, tissues, drugs, and diseases. The data analysis and interpretation in IPA are based on the comprehensive, manually curated, and corrected QIAGEN^®^ Knowledge Base. Utilizing robust algorithms backed by this database, IPA can uncover upstream and downstream regulatory factors, interaction relationships, mechanisms, functions, and pathways corresponding to the analyzed molecular data. This allows for an in-depth understanding of the reasons behind changes in the analyzed datasets. In this study, IPA was employed to conduct a canonical pathway analysis of the core regulatory gene set.

### 4.5. scRNA-Seq Data Preparation

Using the keywords “MCAO”, “ischemic stroke”, and “stroke”, we performed a search in the GEO database, selecting “mouse” as the species. From this search, we downloaded the dataset GSE174574 [35]. scRNA-seq data processing and analysis

To ensure the reliability of analysis, we utilized the Seurat package for processing and analyzing single-cell data. The following criteria were applied for gene filtering and the exclusion of low-quality cells:

1. Cells with detected gene counts >200 and <2500 were retained;

2. Cells with mitochondrial gene content ≤ 10% were retained;

3. Genes expressed in at least three cells were retained.

Additionally, we employed the CellCycle Scoring function in Seurat to assess the expression of cell cycle-related genes in single-cell data.

Following annotation, the Seurat FindAllMarkers function was employed to calculate DEGs across different cell types. Using the Wilcoxon rank-sum test, each cell type was compared against all other cells to identify DEGs specific to each cell type. Genes with *p* < 0.05 and |LOGFC| ≥ 0.25 were considered statistically significant.

Additionally, we conducted inter-group comparisons of the same cell type to identify DEGs specific to the same cell type in the context of cerebral I/R injury.

#### 4.5.1. scRNA-Seq Enrichment Analysis

Using five single-cell scoring methods—AUCell, UCell, singscore, ssGSEA, and Add—we performed enrichment scoring for the target gene set, resulting in a total enrichment score. Furthermore, we performed GO and KEGG enrichment analyses on the DEGs identified earlier, using appropriate R packages.

Additionally, we employed a method based on the “Metabolic landscape of the tumor microenvironment at single-cell resolution” [36] to analyze metabolic conditions at the single-cell level.

##### Cell Communication Analysis

To systematically analyze cell–cell communication networks at the molecular level, we utilized CellChat to investigate ligand–receptor interactions between cells. The minimum number of interacting cells was set to 10.

##### Cell Trajectory Analysis

To understand the differentiation trajectories of cells, we utilized Monocle2 software (2.26.0) for analysis. To arrange cells along this inferred trajectory, we designated a starting point for the differentiation pathway. Finally, using the orderCells function, we sequentially positioned all cells along the established trajectory. This methodology allows us to visualize and analyze the progression of cellular differentiation within the dataset.

##### Cell Subpopulation Reanalysis

Target cell populations were extracted, subjected to dimensionality reduction, and re-clustered for further analysis. This allowed for a detailed examination of the specific cellular subsets of interest, providing deeper insights into their characteristics and functions.

### 4.6. Molecular Docking

To identify potential active compounds in Huanglian Jiedu Decoction, we utilized a combination of database searches and literature review. The databases employed for this search included TCMSP, TCMID, DCABM-TCM, HERB, and SYMMAP. By analyzing these data and cross-referencing with the existing literature, we identified pyroptosis-related genes that are significant in IS and may serve as potential targets.

Small molecule structures were downloaded from the PubChem database, while protein structures were obtained from the PDB database. After downloading the respective structures, we performed preprocessing steps. For the molecular docking studies, we utilized AutoDock software (4.2.6). Post-docking, we used PyMOL for visualizing the docking results.

### 4.7. SPR Affinity Analysis

To prepare the drug samples, we utilized the previously prepared lyophilized powder. This powder was dissolved in ultrapure water to achieve a concentration of 100 mg/mL. The solution was then centrifuged at 12,000 rpm for 10 min at 4 °C. Following centrifugation, the supernatant was collected and filtered through a 0.22 μm membrane to produce the final drug sample.

For the protein-loaded chips, we employed a CM5 chip coupled with NLRP3 protein. Prior to initiating the procedure, a buffer solution was prepared by diluting 10× HBS-EP+ buffer with deionized water to a 1× concentration, resulting in a total volume of 500 mL. This mixture was thoroughly blended and placed in the buffer bottle for use.

Once the materials were prepared and the sample injection speed and duration were set on the instrument, the drug sample was injected. The baseline was allowed to stabilize before executing the inject and recover procedure, which involved target fishing and sample collection. After the procedure, the collected samples were stored at −20 °C for future use.

### 4.8. LC-MS Analysis

To identify small molecules that specifically bind to NLRP3, the solutions binding to both the blank chip and the NLRP3 protein chip were combined. The mixed solutions underwent ultrasonic treatment and were filtered through a 0.22 μm membrane before performing LC-MS analysis.

Chromatography Conditions: Column: ACQUITY UPLC HSS T3 (2.1 × 100 mm, 1.8 μm); Mobile Phase: Acetonitrile solution (A), 0.1% formic acid aqueous solution (B); Gradient Elution:0–6 min: 95% B; 6–24 min: 75% to 65% B; 24–32 min: 65% to 5% B; 32–34 min: 5% B; 34–35 min: 5% to 95% B; 35–40 min: 95% B; Flow Rate: 0.3 mL/min; Column Temperature: 35 °C; Injection Volume: 3 μL.

Mass Spectrometry Conditions: Ionization Mode: Positive and negative ion modes; Ion Source: Heated Electrospray Ionization; Ion Source Temperature: 400 °C; Ionization Source Voltage: 3.5 kV (positive ion mode), 3 kV (negative ion mode); Capillary Temperature: 320 °C; S-Lens Voltage: 55 V; Sheath Gas: High purity nitrogen (>99.99%), flow rate 35 arb; Auxiliary Gas: High purity nitrogen, flow rate 10 arb; Scan Mode: Full MS/dd-MS2; Full MS Resolution: 70,000; dd-MS2 Resolution: 17,500; Scan Range: m/z 100–1500 Da; Collision Energies: 10, 30, 50 eV.

Post-acquisition, data processing was conducted using Thermo Scientific Xcalibur software (OPTON-30967). The results obtained from the blank chip binding served as the background, which was subtracted from the NLRP3-binding data to isolate small molecules specifically binding to NLRP3.

### 4.9. MD

MD simulations were performed using GROMACS (GMX-2024 GPU-CUDA), employing the CHARMM36 force field and the TIP3P water model. The system was confined within a cubic box with a size of 1.2 nm. Solvent molecules were added using the SPC216 water model. To neutralize the system, Na^+^/Cl^−^ ion pairs were added to balance the charge.

To minimize potential collisions between the protein and small molecules, energy minimization was carried out using the steepest descent method to achieve an optimal potential energy state. The system was then equilibrated under isothermal conditions at 300 K, followed by isothermal–isobaric equilibration with Parrinello–Rahman pressure coupling at 1 bar for 1000 ps, using a time step of 2 fs. Coordinates and energy of the system were saved every 10 ps.

Following equilibration, each system underwent a 100 ns simulation. The resulting molecular trajectories were then corrected and evaluated.

To further refine the results from the MD simulations, binding free energy calculations were performed to quantify the affinity between the protein and small molecule ligands. The Molecular Mechanics Poisson–Boltzmann Surface Area (MMPBSA) method was employed to predict the stability of the ligand–receptor complex post-MD simulation.

### 4.10. SPR Molecular Fishing

To investigate the interactions between target proteins and candidate small molecules from HLJD, protein chips were prepared. The test compounds were first dissolved and then diluted to a series of concentrations using appropriate buffer solutions. These diluted solutions were then injected into the flow cells of the chip.

If an interaction between the tested compounds and the target protein occurs, it will be as evident as a binding and dissociation curve. The binding affinity constants, Ka (association rate constant) and Kd (dissociation rate constant), as well as the equilibrium dissociation constant KD, were calculated using Biacore T200 software (CY25775). The equilibrium dissociation constant KD is determined by the ratio Kd/Ka and represents the affinity between the candidate small molecules and their respective target proteins from Huanglian Jiedu Decoction.

### 4.11. RT–qPCR

Approximately 50 mg of brain tissue was weighed and homogenized for total RNA extraction. The same method was used to extract RNA from cells. Following RNA extraction, reverse transcription was performed to generate cDNA templates. RT–qPCR was then conducted using fluorescent probes. The expression levels of the genes of interest were normalized to β-actin, and the relative gene expression was determined using the 2^−ΔΔCt^ method.

### 4.12. WB

WB experiments were conducted using the Bio-Rad TGX Stain-Free FastCast Kit (Hercules, CA, USA). The total protein obtained from the kit served as the reference for subsequent analyses. The following primary antibodies were used: NLRP3 (1:1000), ASC (1:1000), pro Caspase-1 + p10 + p12 (1:1000), and GSDMD (1:1000). The relative protein expression was confirmed by total protein quantification. Protein detection and analysis were performed using Image Lab software (SOFT-LIT-170-9690-ILSPC-V-6-1).

### 4.13. ELISA

The levels of IL-1β and IL-18 in the ischemic cortex of the brain were detected using the ELISA kit for rats following the manufacturer’s instructions. Briefly, the mice were anesthetized and sacrificed, the ischemic cortex was dissected and lysed, the homogenate was centrifuged at 12,000 rpm at 4 °C for 10 min, and then the supernatant was collected for ELISA.

### 4.14. Cytokine/Chemokine Measurement

We measured the levels of cytokines and chemokines in serum and brain. The levels of multiple inflammatory factors were detected by the cytometric bead array system. CXCL1, G-CSG, IFN-γ, IL-1β, IL-2, IL-4, IL-6, IL-10, IL-12p70, IL-17, IL-23p19, MCP-1, and TNF-α were detected in blood. We determined GM-CSF, IFN-β, IFN-γ, IL-1α, IL-1β, IL-6, IL-10, IL-12p70, IL-17A, IL-23, IL-27, MCP-1, and TNF-α.

## 5. Conclusions

**Figure 11:**
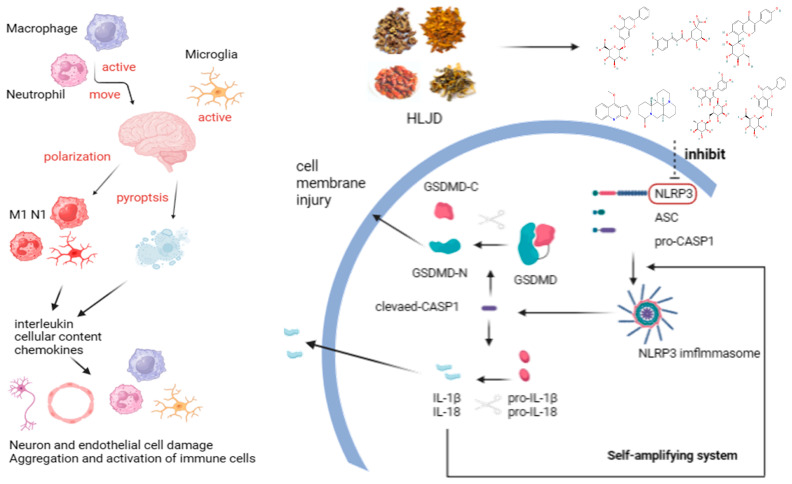
After IS occurs, microglia, macrophages, and neutrophils become activated. Macrophages and neutrophils infiltrate the brain and, along with microglia, polarize into M1 and N1 pro-inflammatory phenotypes. These cells undergo pyroptosis, releasing inflammatory factors, cellular contents, and chemokines, which ultimately damage neurons and endothelial cells, impair neural function, disrupt the BBB, and further activate and recruit immune cells, exacerbating cerebral ischemia. The small molecules in HLJD can bind to NLRP3, inhibiting its function, thus disrupting the self-amplifying cycle of pyroptosis, reducing the production of GSDMD-N and mature IL-1β and IL-18, mitigating the progression of pyroptosis, and providing neuroprotective effects.

During the IS process, the brain’s resident microglia activate, causing neuroinflammation and releasing chemokines to recruit peripheral macrophages and neutrophils, leading to further inflammatory damage. In this process, the classical inflammasome-related pyroptosis pathway in these three cell types is activated and undergoes a self-amplifying cycle, damaging other cells and resulting in severe cerebral ischemia and neurological dysfunction. Compounds in HLJD, such as Chrysin-7-O-Glucuronide, Isomartynoside, Neochlorogenic Acid, Puerarin, Dictamine, Matrine, Rutin, Skullcapflavone II, and Wogonoside, may bind to NLRP3, inhibiting its function. This inhibition can control the self-amplifying classical inflammasome-related pyroptosis pathway, reducing its activation level and decreasing mRNA and protein expression levels, thereby preventing excessive inflammatory damage. Additionally, HLJD can improve the inflammatory microenvironment in both the brain and peripheral blood, reducing pro-inflammatory cytokine levels, increasing anti-inflammatory cytokine levels, and mitigating the occurrence and progression of inflammation and cell pyroptosis (Figure 11).

## Figures and Tables

**Table 1:** Binding energies of the top 10 ligand receptors for molecular docking.

Binding Energy Ranking	Protein Name	Pubchem ID	Binding Energy (kcal/mol)
1	NLRP3	180311	−12.7
2	NLRP3	156766	−12.5
3	NLRP3	119041	−12.4
4	IL-18	3082381	−12
5	NLRP3	9817839	−12
6	NLRP3	5321283	−11.9
7	NLRP3	91472	−11.8
8	NLRP3	91885211	−11.8
9	NLRP3	118701246	−11.7
10	NLRP3	15559351	−11.7

**Table 2:** Binding energy from molecular dynamics MMPBSA calculations.

Ligand	Vander Waals Force/(KJ·mol^−1^)	Electrostatic Potential Energy/(KJ·mol^−1^)	Polar Solvation Energy/(KJ·mol^−1^)	Solvent-Accessibility Surface Area Energy/(KJ·mol^−1^)	Binding Free Energy/(KJ·mol^−1^)
**Chrysin**	**−176.7 ± 1.6**	**−29.8 ± 2.4**	**149.6 ± 2.4**	**−21.0 ± 0.1**	**−78.0 ± 1.3**
**Dictamine**	**−125.4 ± 0.5**	**−16.6 ± 0.4**	**54.9 ± 0.5**	**−12.3 ± 0.0**	**−99.4 ± 0.7**
**Isomartynoside**	**−272.1 ± 1.7**	**−100.9 ± 2.0**	**277.0 ± 2.2**	**−31.9 ± 0.1**	**−127.9 ± 1.9**
**Martrine**	**−149.1 ± 1.1**	**3.2 ± 0.5**	**48.2 ± 1.0**	**−14.9 ± 0.0**	**−112.7 ± 0.9**
**Neochlorogenic** Acid	**−158.1 ± 1.0**	**−40.9 ± 1.7**	**177.1 ± 2.1**	**−19.8 ± 0.1**	**−41.8 ± 1.3**
**Puerarin**	**−198.9 ± 1.3**	**−52.0 ± 2.0**	**197.3 ± 2.5**	**−22.5 ± 0.1**	**−76.2 ± 1.5**
**Rutin**	**−212.3 ± 1.4**	**−84.0 ± 1.9**	**217.0 ± 2.9**	**−23.3 ± 0.1**	**−102.5 ± 1.8**
**Skullcapflavone** II	**−147.6 ± 0.9**	**−20.8 ± 0.7**	**73.1 ± 0.9**	**−15.7 ± 0.1**	**−111.0 ± 1.1**
**Wogonoside**	**−206.4 ± 0.9**	**−32.1 ± 1.5**	**204.8 ± 2.9**	**−22.9 ± 0.1**	**−56.7 ± 1.6**

## Data Availability

The sequencing data came from the GEO database.

## Supplementary Materials

The following supporting information can be downloaded at: https://www.mdpi.com/article/10.3390/ph18060775/s1, Figure S1: Pyroptosis signaling pathway map was analyzed by IPA; Figure S2: Single cell quality, dimension reduction, clustering, to batch graphic; Figure S3: UMAP and TSNE plots of the control group and IS group after dimensionality reduction; Figure S4: Enrichment analysis of differentially expressed genes in microglia, macrophages, and neutrophils between the control group and IS group; Figure S5: Cell communication; Figure S6: Subpopulation analysis of macrophages; Figure S7: Subpopulation analysis of neutrophils; Figure S8: NLRP3 molecular dynamic binding of nine small molecules to RMSF determined by LC-MS; Figure S9: NLRP3 molecular dynamic binding of nine small molecules to RG determined by LC-MS; Figure S10: LRP3 molecular dynamic binding of nine small molecules to H-bone determined by LC-MS; Figure S11: NLRP3 molecular dynamic binding of nine small molecules to SASA determined by LC-MS. Table S1: The pyroptosis gene set contains genes; Table S2: Score for pyroptosis gene expression levels in each cell type; Table S3: Molecular docking uses the protein name with the corresponding; Table S4: Score for pyroptosis gene expression levels in each cell type.

## Abbreviations

ASC, Apoptosis-associated speck-like protein containing a CARD; BBB, blood–brain barrier; CBF, cerebral blood flow; DEGs, differentially expressed genes; EB, Evans blue staining; ELISA, Enzyme-linked immunosorbent assay; GO, Gene Ontology; GSDM, Gasdermin; HLJD, Huanglian Jiedu Decoction; IPA, ingenuity Pathway Analysis; I/R, ischemia/reperfusion; IS, Ischemic stroke; KEGG, Kyoto Encyclopedia of Genes and Genomes; LC-MS, Liquid Chromatography-Mass Spectrometry; MD, Molecular dynamics; MMPBSA, Molecular Mechanics Poisson–Boltzmann Surface Area; NLRP3, NLR family pyrin domain containing 3; PCA, principal component analysis; PPI, protein–protein interaction; scRNA-seq, single-cell RNA sequencing; SPR, surface plasmon resonance; ssGSEA, Single sample gene set enrichment analysis; TCM, traditional Chinese medicine; tMCAO, transient middle cerebral artery occlusion; TTC,2,3,5-triphenyltetrazolium chloride staining; WB, Western blot.
