# Supplementary material for: Huanglian Jiedu Decoction Treats Ischemic Stroke by Regulating Pyroptosis: Insights from Multi-Omics and Drug–Target Relationship Analysis
Source: Pharmaceuticals (Basel). 2025 May 23;18(6):775. doi: 10.3390/ph18060775 (PMC12195757; doi:10.3390/ph18060775)
Supplement: Supplementary file 1 [file pharmaceuticals-18-00775-s001.zip › pharmaceuticals-3583699-supplementary1.pdf]

Supplementary Fig. S1. Pyroptosis signaling pathway map was analyzed by IPA.Green for the data in the data, determine inhibition/downgrade red data to determine activation/hikes, orange data to predict the activation/hikes, blue for cut/data, yellow for doubt need to pay special attention to the data.

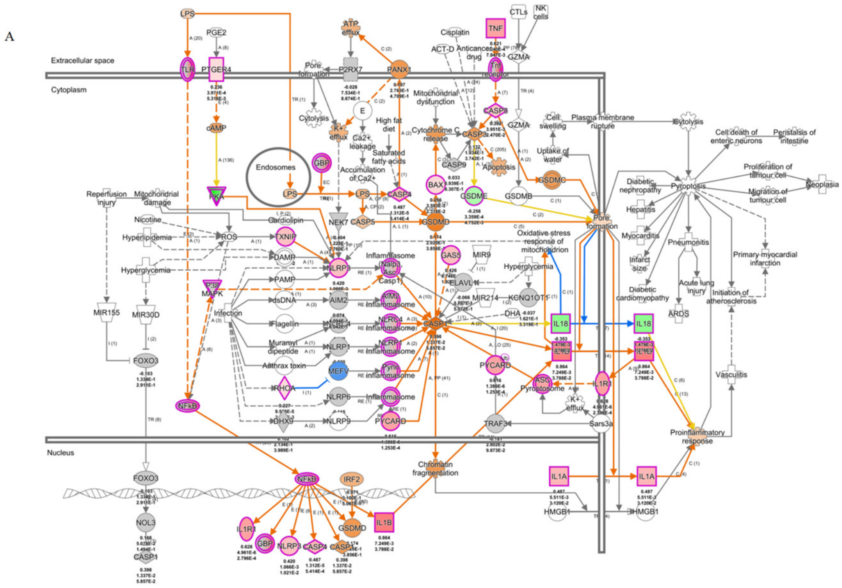

Supplementary Fig. S2. Single cell quality, dimension reduction, clustering, to batch graphic. (A) Data before quality control. (B) Data after quality control. (C) The PC number of elbow plot. (D) Clustering tree diagram. (E) Plot of the batch effect. (F) Plot of the cell cycle.

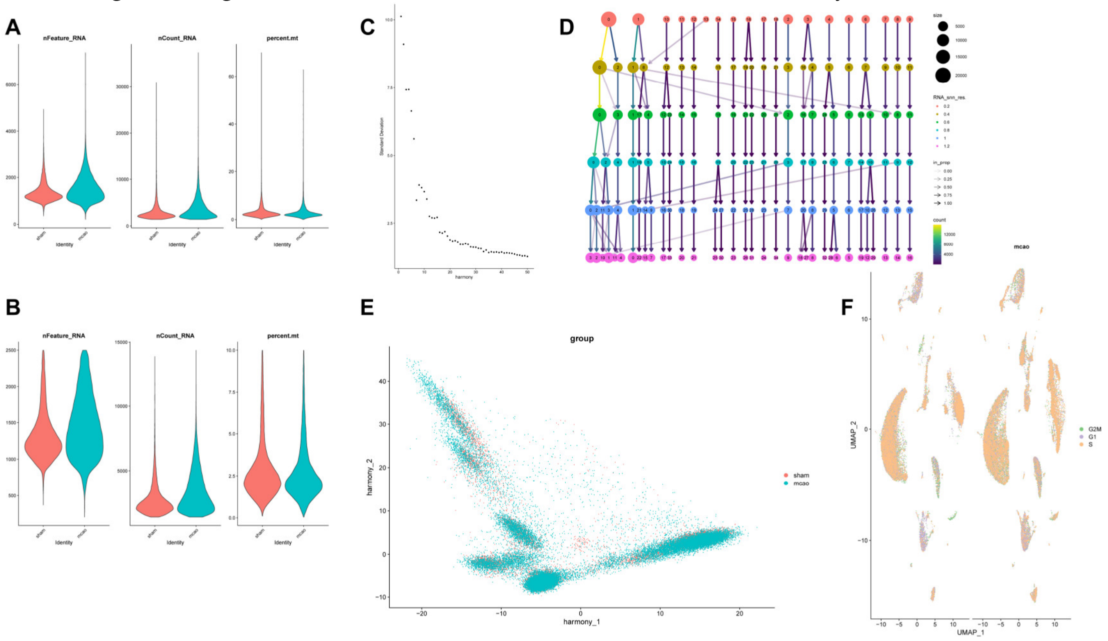

Supplementary Fig. S3. UMAP and TSNE plots of the control group and IS group after dimensionality reduction. (A) UMAP plot of the control group and IS group after dimensionality reduction. (B) T-SNE plot of the control group and IS group after dimensionality reduction.

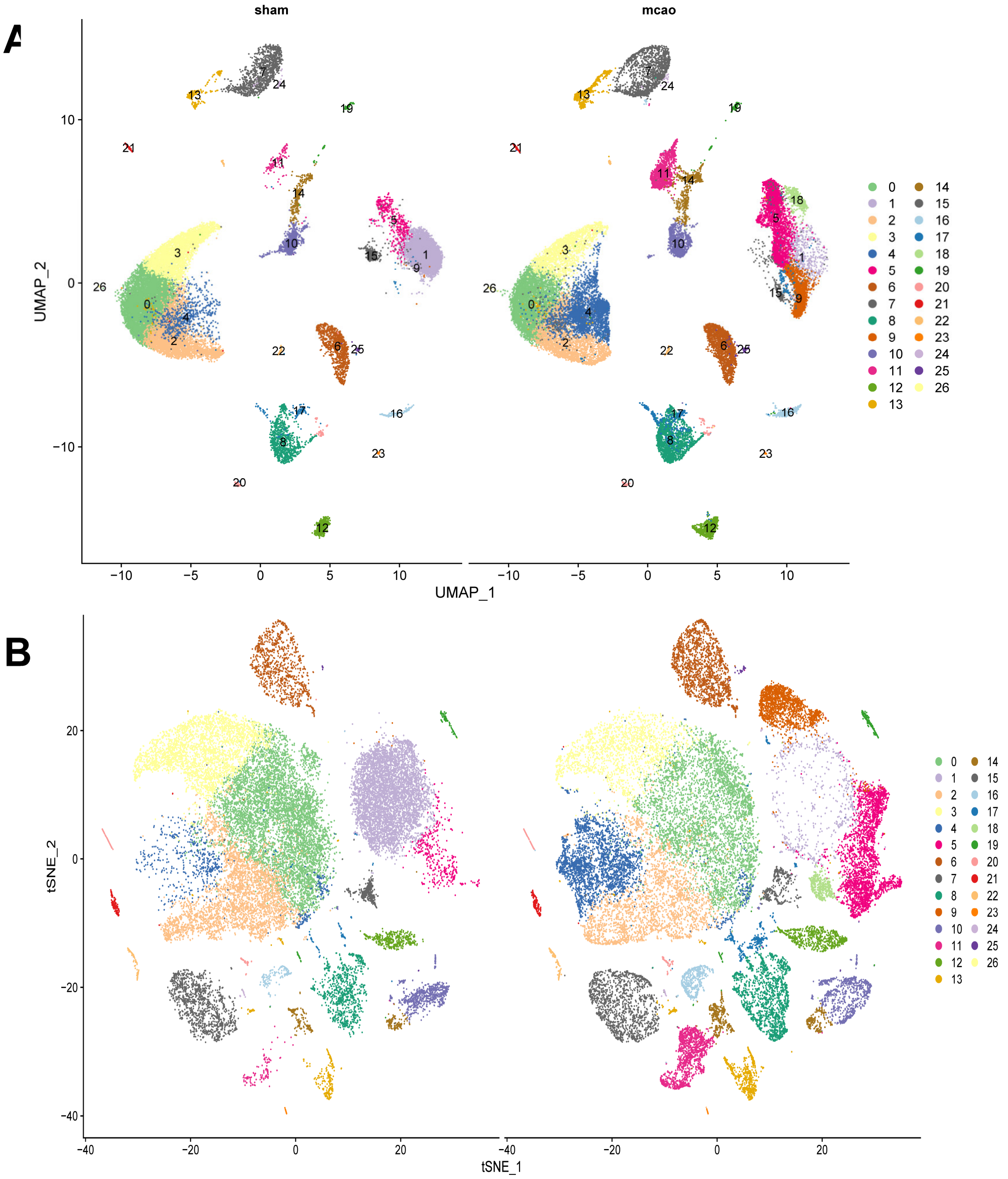

Supplementary Fig. S 4. Enrichment analysis of differentially expressed genes in microglia, macrophages, and neutrophils between the control group and IS group. (A) GO analysis of DEGS in microglia. (B) KEGG analysis of DEGS in microglia. (C) GO analysis of DEGS in macrophages. (D) KEGG analysis of DEGS in macrophages. (E) GO analysis of DEGS in neutrophils. (F) KEGG analysis of DEGS in neutrophils.

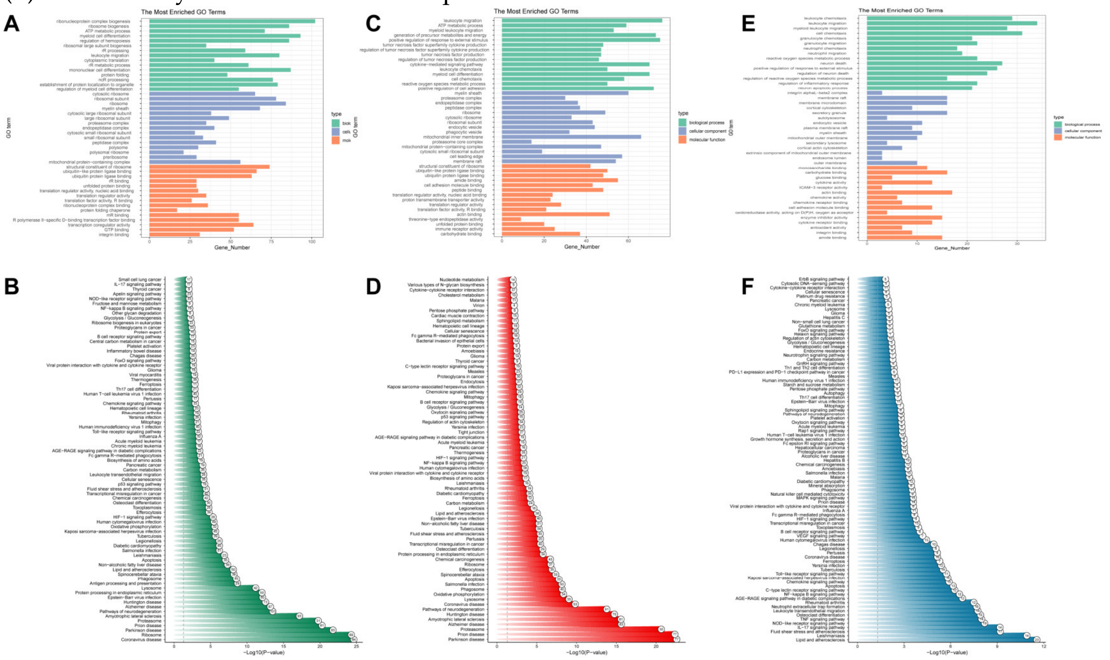

Supplementary Fig. S5. Cell communication. (A) Source cell communication relationship. (B) Control group cell communication strength. (C) Control group cell communication number. (D) IS group cell communication strength. (E) IS group cell communication number. (F) Each cell in the contral group as the strength of the specific ligand receptor. (G) Each cell in the IS group as the strength of the specific ligand receptor.

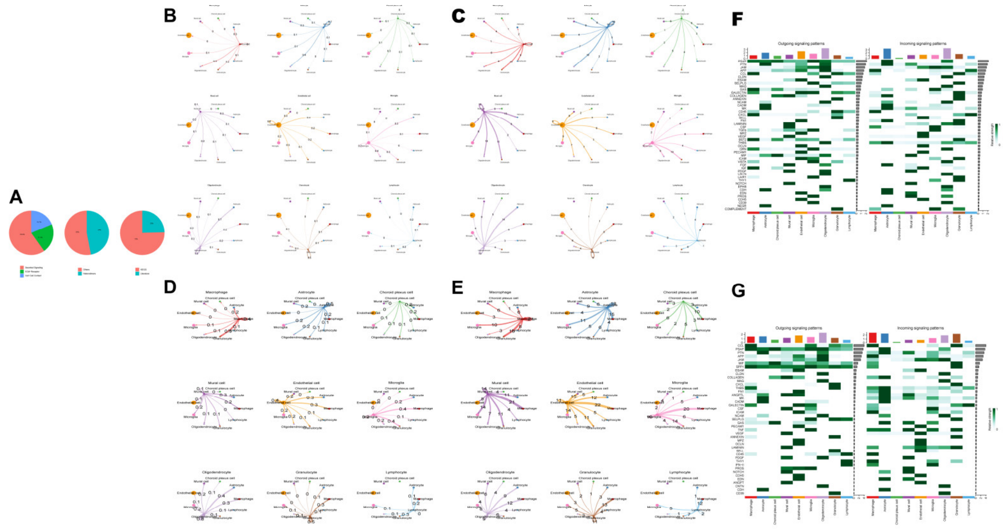

Supplementary Fig. S6. Subpopulation analysis of macrophages. (A) Pyroptosis gene set scores in subtypes of macrophages.(B) Proportion of the number of macrophages subsets in each group. (C) The ratio of P- macrophages and P+ macrophages in sham group and mcao group. (D) Quasi-temporal analysis of macrophages arranged on a quasi-temporal axis

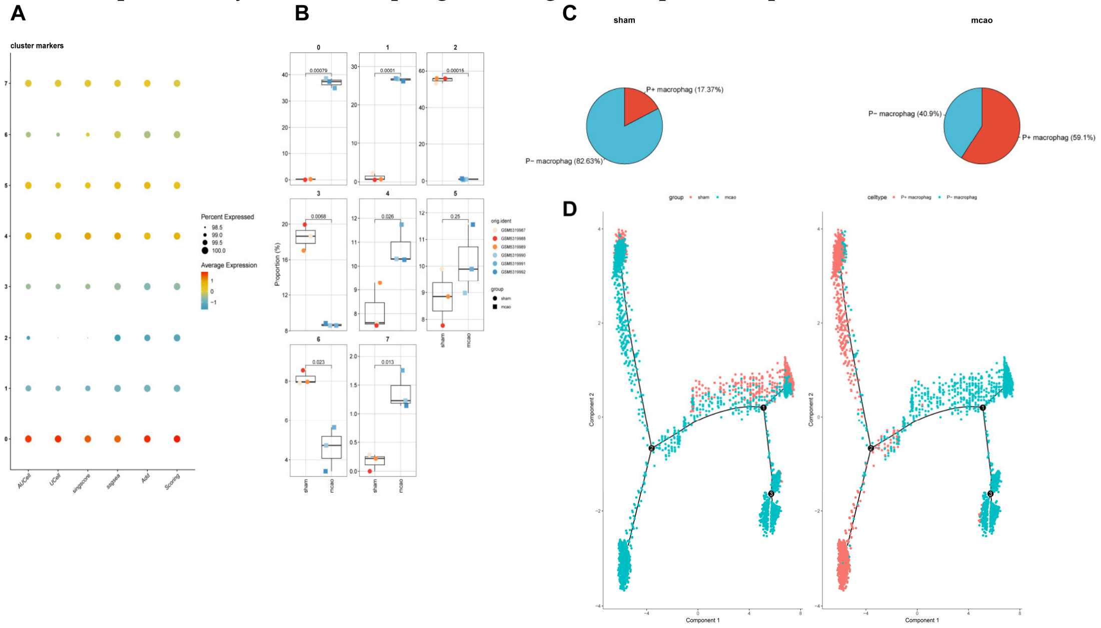

Supplementary Fig. S7. Subpopulation analysis of neutrophils. (A) Pyroptosis gene set scores in subtypes of neutrophils.(B) Proportion of the number of neutrophils subsets in each group. (C) The ratio of P- neutrophilsand P+ neutrophilsin sham group and mcao group. (D) Quasi-temporal analysis of neutrophils arranged on a quasi-temporal axis

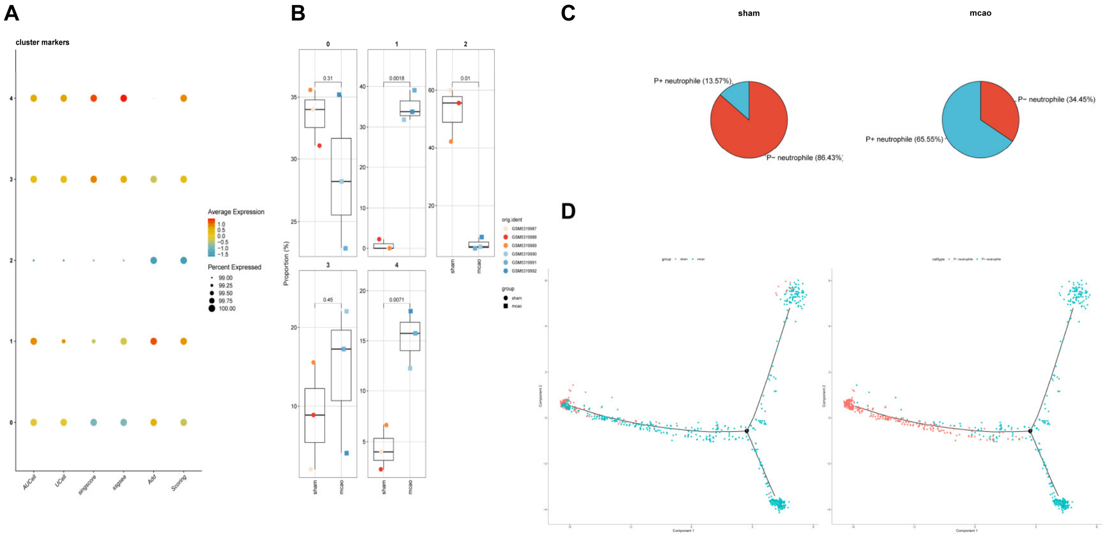

Supplementary Fig. S8. NLRP3 molecular dynamic binding of nine small molecules to RMSF determined by LC-MS. (A) RMSF of NLRP3-Chrysin-7-O-Glucuronide. (B) RMSF of NLRP3-Dictamine. (C) RMSF of NLRP3-Isomartynoside. (D) RMSF of NLRP3-Matrine. (E) RMSF of NLRP3-Neochlorogenic Acid. (F) RMSF of NLRP3-Puerarin. (G) RMSF of NLRP3-Rutin. (H) RMSF of NLRP3-Skullcapflavone II. (I) RMSF of NLRP3-Wogonoside

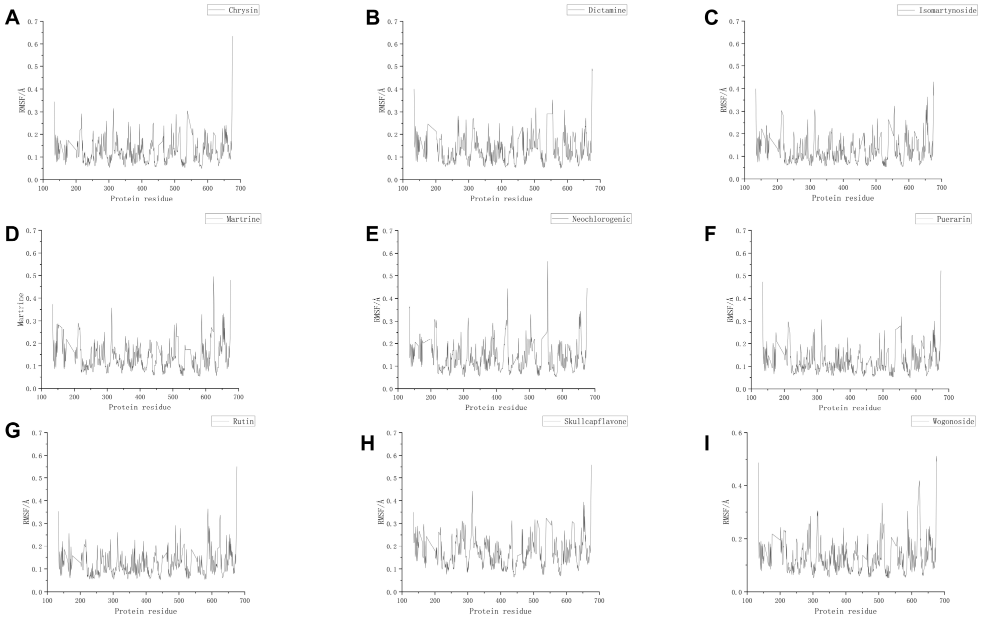

Supplementary Fig. S9. NLRP3 molecular dynamic binding of nine small molecules to RG determined by LC-MS. (A) RG of NLRP3-Chrysin-7-O-Glucuronide. (B) RG of NLRP3-Dictamine. (C) RG of NLRP3-Isomartynoside. (D) RG of NLRP3-Matrine. (E) RG of NLRP3-Neochlorogenic Acid. (F) RG of NLRP3-Puerarin. (G) RG of NLRP3-Rutin. (H) RG of NLRP3-Skullcapflavone II. (I) RG of NLRP3-Wogonoside.

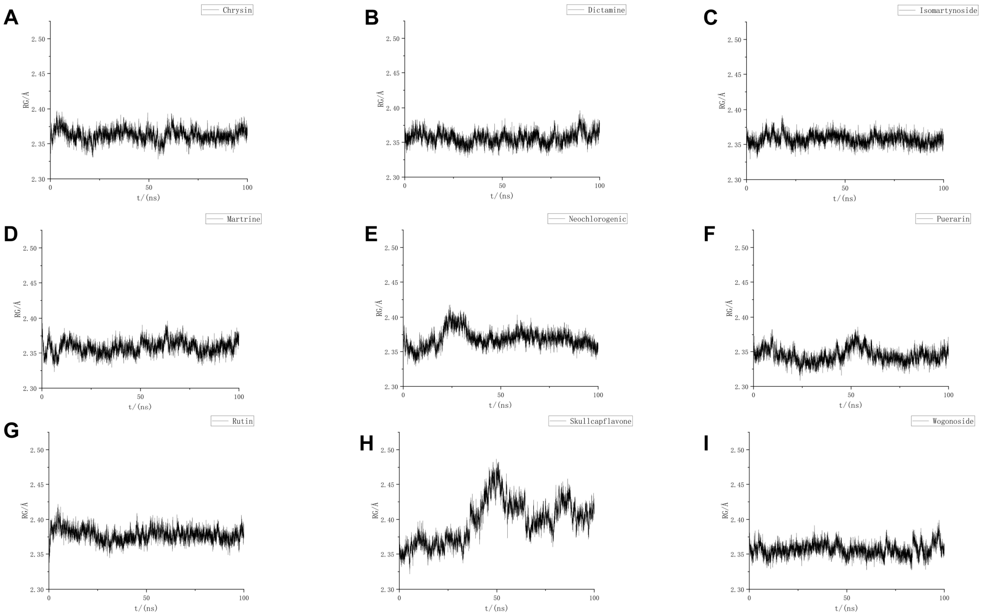

Supplementary Fig. S10. NLRP3 molecular dynamic binding of nine small molecules to H-bone determined by LC-MS.

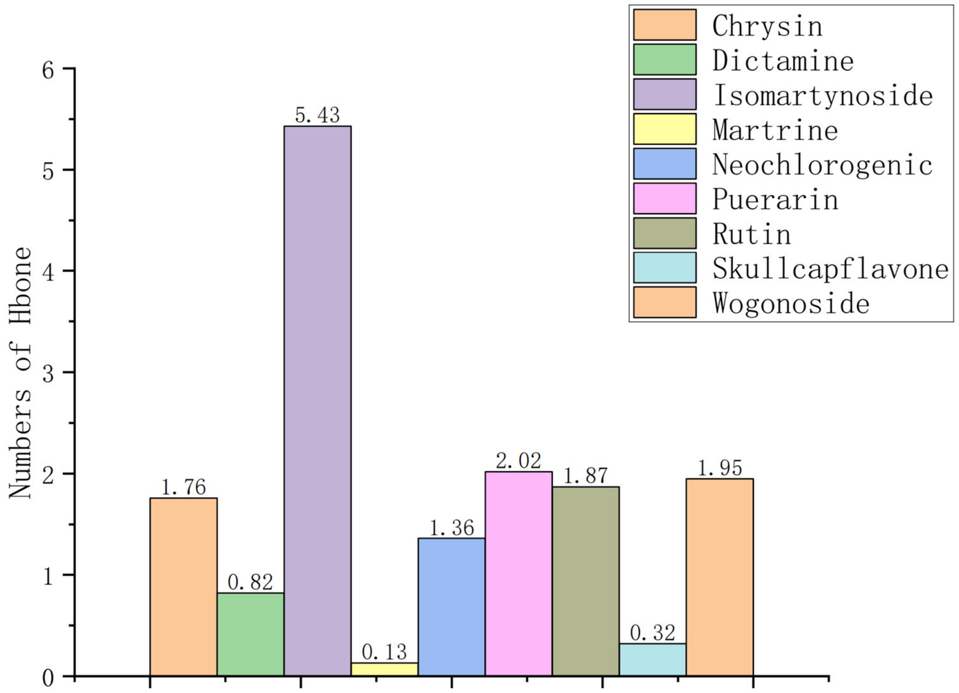

Supplementary Fig. S 11. NLRP3 molecular dynamic binding of nine small molecules to SASA determined by LC-MS. (A) SASA of NLRP3-Chrysin-7-O-Glucuronide. (B) SASA of NLRP3-Dictamine. (C) SASA of NLRP3-Isomartynoside. (D) SASA of NLRP3-Matrine. (E) SASA of NLRP3-Neochlorogenic Acid. (F) SASA of NLRP3-Puerarin. (G) SASA of NLRP3-Rutin. (H) SASA of NLRP3-Skullcapflavone II. (I) SASA of NLRP3-Wogonoside.

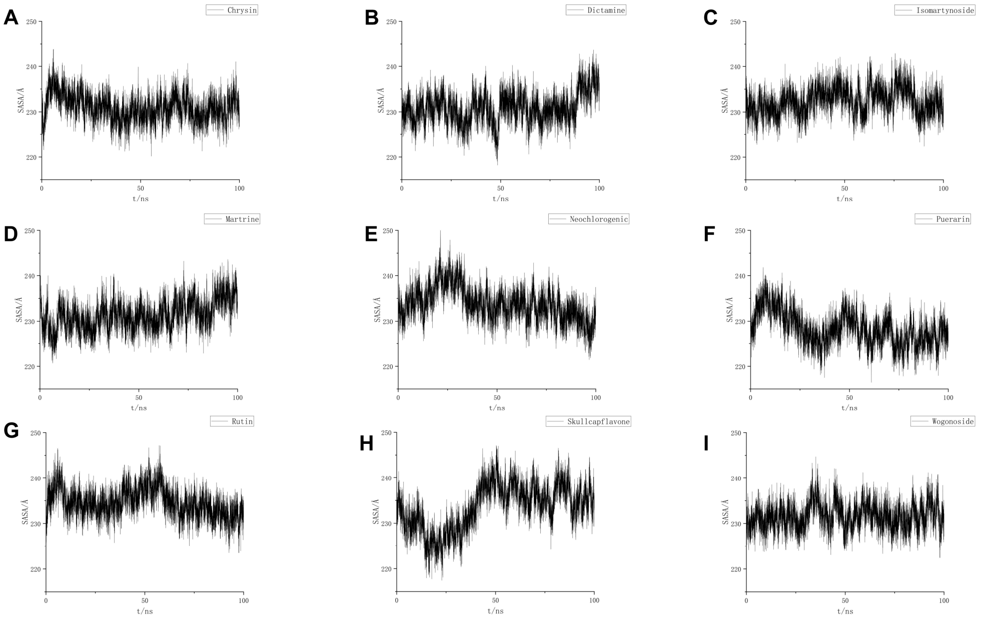

Supplementary tables S1 The pyroptosis gene set contains genes

Gene Symbol
Bak1
Bax
Caspase-1
Caspase-3
Caspase-4
Caspase-8
Chmp2a
Chmp2b
Chmp3
Chmp4b
Chmp4c
Chmp6
Chmp7
Cycs
Elane
Gsdmd
Gsdme
Hmgb1
Il1a
Il1b
Il1r1
Irf2
Nlrp3
Pycard（ASC）

Supplementary tables S2 Score for pyroptosis gene expression levels in each cell type

Celltype	Mean scroe	Median score
Endothelial cell	1.503385542	1.489418003
Macrophage	2.067013329	2.059372409
Astrocyte	1.268651351	1.260894119
Microglia	1.718862379	1.708088754
Oligodendrocyte	1.46712627	1.464135929
Mural cell	1.488209642	1.468885103
Lymphocyte	1.589901416	1.587018508
Granulocyte	1.889564	1.881701926
Choroid plexus cell	1.423680435	1.427696992

Supplementary tables S3 Molecular docking uses the protein name with the corresponding IDENTIFIER.

**Protein name**	IDENTIFIER
NLRP3	8WSM
ASC	2KN6
Caspase-1	5FNA
Caspase-3	1NME
Caspase-4	6NRY
Caspase-8	5JQE
IL-1β	6Y8I
IL-18	3OW4
GSDMD	6AO4
