# Supplementary material for: Huanglian Jiedu Decoction Treats Ischemic Stroke by Regulating Pyroptosis: Insights from Multi-Omics and Drug–Target Relationship Analysis
Source: Pharmaceuticals (Basel). 2025 May 23;18(6):775. doi: 10.3390/ph18060775 (PMC12195757; doi:10.3390/ph18060775)
Supplement: Supplementary file 1 [file pharmaceuticals-18-00775-s001.zip › pharmaceuticals-3583699-supplementary2.pdf]

Supplementary Tables S4. Score for pyroptosis gene expression levels in each cell type.

Name	Formula	Annot. Source: Predicted Compositions	Annot. Source: mzCloud Search	Annot. Source: ChemSpider Search	Annot. Source: MassList Search	Annot. DeltaMass [ppm]	Calc. MW	m/z	RT [min]	Area (Max.)	ChemSpider Results	mzCloud Results	mzCloud Best Match	mzCloud Best Match Confidence	mzCloud Best Tree Match	Mass List Match: import
Matrine	C15 H24 N2 O	Full match	No results	Partial match	Full match	-0.57	248.18872	249.196	2.422	31469240.28	160	Single match found	No MS2	[M+H]+1	31469240.28	8.4
Dictamine	C12 H9 N O2	No results	No results	Not the top hit	Full match	-1.45	199.06304	200.07032	19.388	13967657.39	85	Single match found	No MS2	[M+H]+1	13967657.39	6.2
Skullcapflavone Ii	C19 H18 O8	No results	No results	Partial match	Full match	2.2	374.10099	375.10827	27.423	8019867.312	163	Multiple matches found	No MS2	[M+H]+1	8019867.312	6.2
Wogonoside	C22 H20 O11	No results	No results	Partial match	Full match	0.54	460.10081	461.1077	12.572	7341007.697	99	Multiple matches found	No MS2	[M+H]+1	7341007.697	6.2
Rutin	C27 H30 O16	No results	No results	Full match	Full match	0.8	610.15387	633.14305	7.944	1857563.843	74	Single match found	No MS2	[M+Na]+1	1857563.843	6.2
